# Linking Nutrients to Multiple Sclerosis Pathogenesis: Biological Evidence and Clinical Implications

**DOI:** 10.3390/nu17213414

**Published:** 2025-10-30

**Authors:** Rachele Rosso, Alessandro Maglione, Matteo Bronzini, Eleonora Virgilio, Marinella Clerico, Simona Rolla

**Affiliations:** 1 Department of Clinical and Biological Sciences, University of Turin, 10043 Orbassano, Italy; rachele.rosso@unito.it (R.R.); alessandro.maglione@unito.it (A.M.); eleonora.virgilio@unito.it (E.V.); marinella.clerico@unito.it (M.C.); 2 Department of Computer Science, University of Turin, 10149 Torino, Italy; 3 San Luigi Gonzaga University Hospital, 10043 Orbassano, Italy; matteo.bronzini@unito.it

**Keywords:** macronutrients, multiple sclerosis, gut microbiota

## Abstract

Multiple Sclerosis (MS) is an autoimmune and neurodegenerative disorder of the central nervous system (CNS), characterized by demyelination, neuronal loss and physical disability. To date, the exact causes of MS remain unknown. Lifestyle factors, in particular diet, have received growing attention due to their impact on human health, their role in modulating disease pathogenesis, and their influence on gut microbiota composition and activity. As a result, numerous studies have been conducted to examine how specific nutrients, and thereby distinct dietary patterns, may affect the onset and progression of MS. In this narrative review, we aim to explore the most recent and updated evidence concerning the role of fatty acids, carbohydrates, proteins and fibers macronutrients in MS development and progression by evaluating the most relevant literature findings from preclinical models, and clinical trials on people with MS. Dietary macronutrients influence MS pathology through immune and gut–brain axis modulation. Diets rich in saturated fats and refined carbohydrates exacerbate neuroinflammation, promote Th1/Th17 polarization, and worsen disease severity. Conversely, monounsaturated and omega-3 polyunsaturated fatty acids, dietary fibers, and adequate tryptophan metabolism exert anti-inflammatory effects, enhance regulatory T cell (Treg) activity, and improve clinical outcomes. Fiber-derived short-chain fatty acids (SCFAs) and omega-3 metabolites also support gut barrier integrity and suppress astrocyte activation. Evidence on dairy, meat and gluten remains inconclusive, though certain milk proteins and certain components of red/processed meat and of wheat may promote inflammation. Overall, anti-inflammatory and fiber-rich diets, such as those emphasizing unsaturated fats and low sugar intake, appear to confer protective effects in MS. The clarification of the role of dietary components in relation to the disease could help to guide patients toward a healthy and balanced diet, with positive effects on their overall health.

## 1. Introduction

### 1.1. Pathogenesis and Clinical Phenotypes of Multiple Sclerosis

Multiple sclerosis (MS) is a chronic, autoimmune disorder of the central nervous system (CNS), characterized by demyelination and neurodegeneration and consequent physical disability. The average age of onset ranges between 18 and 50 years, and the disease affects approximately 2.9 million individuals worldwide, predominantly women, with a female-to-male ratio of 3:1 [1,2].

MS clinical manifestations result from the formation of plaques within the CNS, accompanied by demyelination, axonal damage and loss, and inflammation, which are hallmarks of the disease. This pathological process occurs as a result of immune cells becoming autoreactive and acquiring the ability to migrate, cross the blood–brain barrier (BBB), and infiltrate the CNS, where they initiate an immune attack. The main cell types involved in this mechanism include CD4^+^ T helper (Th) 1 cells, Th17 cells, CD8^+^ T cells, and B cells. Additionally, cytokines produced by these autoreactive T cells, such as interferon-gamma (IFN-γ), interleukin-17 (IL-17), and granulocyte-macrophage colony-stimulating factor (GM-CSF), contribute to the pathophysiology of the disease [3].

Different clinical forms are now recognized and vary in their pattern of disease progression. The most common form, Relapsing–Remitting MS (RRMS), affects approximately 85% of patients and is characterized by alternating periods of relapse, marked by episodes of neurological dysfunction, and remission, during which clinical stability is maintained and no new neurological symptoms appear [4]. Other forms of MS include Secondary Progressive MS (SPMS), and Primary Progressive MS (PPMS) [4]. SPMS often develops as a consequence of untreated or long-standing RRMS and is phenotypically defined by gradual disability progression that may occur with or without superimposed relapses [4]. In contrast, PPMS is characterized by continuous neurological deterioration from disease onset, without an initial RR phase, although periods of relative stability and occasional relapses can still occur [4]. Additionally, MS can be diagnosed in individuals younger than 18 years, a condition referred to as pediatric MS (PMS), which accounts for approximately 3–10% of all MS cases [5].

Currently, there is no cure for MS, but several disease-modifying therapies (DMTs) are available, which aim to control the symptoms and the disease progression over time [1].

So far, the causes of MS remain unknown; however, it is believed that a combination of genetic and environmental factors may contribute to its onset. Among the environmental factors, research has focused on the potential role of viral infections, such as Epstein–Barr virus (EBV), as well as pollution, vitamin D levels, smoking, and diet [6]. Diet, in particular, has received considerable attention due to its impact on human health and disease pathogenesis.

### 1.2. Nutrition and MS Pathogenesis

The first evidence linking diet to MS dates back to the 1950s, when Dr. Roy Swank observed a lower incidence of MS in coastal regions of Norway, where dietary habits were characterized by a higher intake of polyunsaturated fatty acids and lower consumption of saturated fats [7]. Building on these observations, Dr. Swank conducted a longitudinal study involving 146 MS patients who were advised to follow a low-fat diet in which saturated fats were replaced with unsaturated vegetable oils. He reported that patients who adopted this dietary intervention early after diagnosis, that in those years means before the onset of significant disability, exhibited a high rate of clinical stability, with many remaining unchanged for up to 20 years [8]. However, it should be noted that the first diagnostic criteria for MS were developed in 1954 by Allison and Millar, followed by those proposed by Schumacher in 1965, McAlpine in 1972, Rose in 1976, McDonald and Halliday in 1977, and Poser in 1983 [9]. Before the advent of magnetic resonance imaging (MRI) in the late 1980s, MS diagnosis relied mainly on clinically confirmed relapses, neurophysiological tests (evoked potentials), and cerebrospinal fluid analysis. A definitive diagnosis could take years to establish after the initial onset of symptoms. Plus, aquaporin 4 and MOG auto-antibodies had not yet been discovered, posing a substantial risk of error in the differential diagnosis. In 1997 MRI was finally introduced in the Barkhof MRI diagnostic criteria marking a decisive turning point and allowing earlier and more accurate diagnoses [9,10,11]. Finally in 2001 the concept of dissemination in time (DIT) and space (DIS) were introduced in the McDonald diagnostic criteria, further allowing an increase in sensitivity and specificity. Since then, further updates have been introduced, leading up to the 2024 criteria. The evolution in MS diagnosis needs to be taken into account, particularly when considering studies prior to Barkhof MRI criteria and later McDonald where the concept of “no better explanation” was introduced. Although the Swank study had methodological limitations by current standards, it pioneered the concept that dietary factors might influence MS progression, laying the groundwork for subsequent research.

Further support for a connection between diet and MS arises from the temporal association observed between shifts in disease prevalence and the nutritional transition that has taken place in some parts of the world, notably in China. In 2019, the prevalence of MS in China had doubled compared with 1990, with an age-standardized prevalence rate of 2.32 per 100,000 representing a 23.31% increase since 1990, most of which occurred after 2010 [12]. During this period, China underwent a profound nutritional transition [13], characterized by a substantial increase in the consumption of edible oils across all social classes. Between 1989 and 1991, edible oil intake nearly doubled, while the average per capita intake rose from 23.2 to 41.6 g per day (209 to 374 kilocalories) among adults [14]. Alongside the higher consumption of dietary fats, there was also a marked rise in the intake of pork, beef, chicken, and eggs. Consequently, the traditional Chinese diet, rich in vegetables and carbohydrates and low in animal products, was progressively replaced by a higher-fat dietary pattern, with more than 30% of total energy derived from fat. In 2006, it was estimated that two-thirds of adults (67.0%) were consuming diets in which over 10% of total energy came from animal-derived fats, predominantly saturated [13].

Globally, the highest prevalence of MS in 2020 was reported in America (117.49 per 100,000 population) and Europe (142.81 per 100,000 population) [15], regions characterized by higher per capita consumption of red and processed meat, sugar-sweetened beverages, and ultra-processed foods in general [16,17]. The progressive westernization of dietary habits, marked by increased intake of convenience and processed foods and a decline in home cooking and traditional culinary practices, has contributed to the rising prevalence of obesity over the past five decades [18]. Obesity, in turn, appears to be associated with the pathogenesis of autoimmune diseases, including MS [19]. During obesity development, adipocytes become hypertrophic, leading to alterations in the resident immune cells that contribute to the development of an inflammatory environment. Adipose tissue and infiltrating immune cells secrete various pro-inflammatory cytokines, such as IL-17, IL-6, and TNF-α, all of which have been implicated in MS pathogenesis [20]. Moreover, adipocyte hypertrophy alters the secretion profile of adipokines that modulate immune function, including leptin, resistin, visfatin, adiponectin, and apelin [21]. Higher levels of pro-inflammatory adipokines (leptin, resistin, visfatin) and reduced levels of anti-inflammatory adipokines (adiponectin, apelin) have been reported in people with MS (pwMS) compared with healthy controls [21].

Further evidence for the link between obesity and MS comes from genetic and epidemiological studies. A significant genetic correlation has been observed between body mass index (BMI) and MS, suggesting that genetic factors play an important role in obesity and MS comorbidity. In particular, the Gametogenetin-Binding Protein 2 (GGNBP2) gene, whose expression is significantly altered in tissues from both obese and MS patients compared with controls, has been identified as a potential common functional gene [22]. A recent meta-analysis reported that overweight and obese individuals had an increased risk of developing MS, with relative risks (RRs) of 1.39 and 1.88, respectively, and a significantly higher risk among obese compared to overweight individuals [23]. Consistent findings from two independent cohort studies have shown that higher BMI at ages 18–20 is associated with an increased likelihood of MS onset later in life, with a twofold risk observed among individuals classified as obese compared with those of normal weight [23]. Furthermore, childhood obesity has been associated with a higher risk of both pediatric and adult-onset MS, particularly among girls [24,25]. Gender-specific analyses indicate that females have a greater risk (RR 2.22) compared with males (RR 1.54), with a statistically significant difference between the two groups [26].

### 1.3. Gut Microbiota and Diet in MS

Another shared feature between obesity and autoimmune diseases such MS is the disruption of gut microbiota homeostasis, a condition known as dysbiosis [27,28]. Diet is among the main factors influencing the composition and functionality of the gut microbiota [29,30,31]. The last comprises a complex community of microorganisms inhabiting the intestinal tract that can interact bidirectionally with the immune system and the CNS through the gut–brain axis. Dietary components provide substrates for microbial metabolism, thereby influencing both microbial activity and community structure, and can also alter intestinal pH, which may facilitate or hinder the colonization of specific microorganisms [29,32]. Given that diet is a modifiable factor, numerous studies have investigated how individual foods and overall dietary patterns may influence the development and progression of MS [33] with the aim to identify dietary strategies that could serve as supportive interventions, potentially enhancing the efficacy of current DMTs for MS.

In this review, we summarize the most recent evidence on the role of macronutrients—including fatty acids, carbohydrates, proteins, and dietary fibers—in the onset and progression of MS. We discuss findings derived from experimental autoimmune encephalomyelitis (EAE) models, as well as current evidence on how these dietary components modulate the gut microbiota. Finally, clinical studies exploring the effects of specific dietary interventions in MS are also reviewed. Although numerous studies have investigated the role of diet in both EAE and MS, as well as the influence of the gut microbiota, few have integrated the evidence regarding the specific impact of individual nutrients on disease development and progression, and their modulation of the gut microbiota. The aim of this review is therefore to provide an updated analysis of the topic, moving beyond the evaluation of specific dietary patterns and highlighting how individual nutrients may shape the gut microbiota, thereby contributing to disease modulation and clinical outcomes.

## 2. Methods

This narrative review includes data from scientific papers collected across several databases, including PubMed, Google Scholar, ScienceDirect, Scopus and Web of Science. The search was aimed at identifying relevant studies regarding the impact of nutrients, fatty acids, carbohydrates, fibers, and proteins on EAE and MS, considering also their effect on the gut microbiota. Therefore, specific keywords were used in the research, such as “nutrients”, “fatty acids”, “carbohydrates”, “fibers” and “proteins”, which were combined with terms like “experimental autoimmune encephalomyelitis”, “multiple sclerosis” and “gut microbiota”, using Boolean operators (AND, OR) to refine the results. The final research was carried out in October 2025, providing an updated overview of the literature. Inclusion criteria included original articles and reviews relevant to the review topic. The research started with a review of the titles of key references, followed by an accurate examination of the content. Articles whose topics were in line with the review subjects were included, while articles that did not satisfy these criteria were excluded. As this is a narrative review, detailed documentation of the literature searches across specific platforms is not required.

## 3. Dietary Fatty Acids

Fatty acids (FAs) represent a family of molecules that are either derived from dietary sources or produced by intestinal bacteria through the fermentation of dietary fibers. Structurally, they consist of a hydrocarbon chain with a hydrophilic terminal carboxyl group. FAs are commonly classified based on the length of their carbon chain and the number of double bonds [31]: short-chain fatty acids (SCFAs) contain 1–5 carbon atoms, medium-chain fatty acids (MCFAs) 6–12, long-chain fatty acids (LCFAs) 13–24, and very-long-chain fatty acids (VLCFAs) more than 24. According to the degree of unsaturation, they can be divided into saturated (SFAs) and mono- or polyunsaturated fatty acids (MUFAs and PUFAs, respectively) [34].

The impact of FAs on MS pathophysiology appears to depend largely on their chemical structure. Lauric acid (LAc; a MCFA) and palmitic acid (PALM; a LCFA) are two representative examples. LAc is mainly found in coconut oil, whereas PALM is abundant in both animal- and plant-derived foods such as butter, milk, chocolate, and eggs. In vivo studies using the EAE model demonstrated that diets enriched in LAc or PALM promote the differentiation of pro-inflammatory Th1 and Th17 cells, worsening the disease course. Specifically, a diet rich in LAc induced an increase in Th17 cells in both the spleen and CNS, suggesting a differential segregation pattern of antigen-specific T cells. Conversely, treatment with SCFAs enhanced the differentiation of regulatory T cells (Tregs). These effects appear to arise from multiple, coordinated mechanisms acting at receptor, transcriptional, and post-transcriptional levels. The gut microbiota seems to play a pivotal role in mediating these effects: fecal microbiota transplantation from mice fed with LAc-rich diet led to an increased abundance of Th17 cells in recipient animals compared with microbiota transplantation from mice fed with control diet. This result indicates that LCFAs induce a perturbation in the gut microbiome and metabolites that act on the immune system [35]. Supporting this evidence, serum concentrations of butyric acid (BA; an SCFA) were found to be decreased, while those of caproic acid (CA; an MCFA) were increased in pwMS. CA levels were positively correlated with Th1 cell abundance, whereas the BA/CA ratio correlated positively with Tregs and negatively with Th1 cells. Hence, an altered SCFA/MCFA ratio may contribute to the chronic inflammatory state associated with MS, paralleling compositional shifts in the gut microbiota [36]. Specifically, a reduction in SCFA-producing taxa (e.g., *Roseburia*, *Coprococcus*, *Blautia*, *Parabacteroides*, and members of the *Lachnospiraceae* family) and an increased abundance of mucin-degrading, pro-inflammatory genera such as *Akkermansia*, *Collinsella*, and *Eubacterium* have been observed in pwMS compared with healthy controls. The role of SCFAs as microbiota-derived metabolites and their immunomodulatory properties will be further discussed in Section 5.

The main dietary MUFA is oleic acid (OA), which represents the predominant lipid component of the Mediterranean diet (MD), largely derived from extra virgin olive oil (EVOO) [37]. EVOO is characterized by its high OA content and a rich profile of antioxidant compounds, particularly phenolic molecules, which contribute to its well-documented health benefits [38]. Although studies investigating the effects of EVOO in the EAE model are relatively recent and limited in number [39,40], emerging findings suggest its protective role. Administration of EVOO, as well as its main components OA and hydroxytyrosol, has been shown to improve clinical outcomes, decrease lipid peroxidation and protein carbonylation, and enhance the activity of antioxidant enzymes such as glutathione peroxidase in the brain, spinal cord, blood, and intestine. Moreover, anti-inflammatory effects of EVOO could result in reduced levels of TNF-α, NF-κB p65, and nitric oxide (NO). Notably, both serum and intestinal lipopolysaccharide (LPS) concentrations were significantly lowered following EVOO or component administration, suggesting that part of its beneficial action may involve modulation of the gut barrier integrity and microbiota composition [39,40].

PUFAs are classified into two main families, omega-3 (ω-3) and omega-6 (ω-6), based on the position of the first double bond from the methyl end of the carbon chain. Specifically, ω-3 FA have their first double bond at the third carbon, whereas ω-6 FA have it at the sixth position. Linoleic acid (LA, a member of ω-6) and α-linolenic acid (ALA; member of ω-3) are defined as essential fatty acids because they cannot be synthesized de novo in humans and must therefore be obtained from the diet. Both serve as precursors for the biosynthetic elongation and desaturation cascade leading to very-long-chain PUFAs.

A high dietary intake of ω-6 is a hallmark of the Western diet, which is typically rich in processed foods and red meat while being poor in fish-derived nutrients. Most seed and vegetable oils (including safflower, sunflower, soybean, maize, grape seed, and cottonseed oils) represent major dietary sources of ω-6 PUFAs in the form of LA, whereas ω-3 fatty acids, particularly ALA, are generally present in lower proportions. Since the human body cannot interconvert ω-6 and ω-3 series fatty acids, tissue concentrations of these FAs and their eicosanoid derivatives, eicosapentaenoic acid (EPA) and docosahexaenoic acid (DHA), depend directly on dietary intake.

Unlike ω-6 FA, the intake of ω-3 is usually insufficient due to limited sources. ALA is found in green leafy vegetables, flaxseed, walnuts, soya and canola oils. Their derivatives, EPA and DHA, are obtained through breast milk and fish oils, such as salmon, mackerel, sardines, anchovies, herring and rainbow trout, but also algae [41]. Epidemiological data indicate an inverse association between ALA intake and MS risk [42]. Furthermore, regular fish or seafood consumption, at least once per week or once per month combined with fish oil supplementation, was associated with a 44% reduction in the odds of MS or clinically isolated syndrome (CIS) compared to lower intake and no supplementation [43]. PwMS who consumed fish three or more times per week or took high-dose ω-3 supplements exhibited lower disability scores, better mobility, improved health-related quality of life, and reduced levels of inflammatory cytokines and matrix metalloproteinase-9 [44,45,46]. In the mouse model, both EPA and DHA have been shown to attenuate EAE disease severity [47,48]. Mechanistically, ω-3 fatty acids appear to modulate T cell activation through GPR120 receptor engagement, leading to decreased phosphorylation of transforming growth factor β-activated kinase 1 (TAK1) and activation of NF-κB, that acts as a T-cell activating mediator [49]. Additionally, DHA has been reported to exert anti-inflammatory and tolerogenic effects by inducing a tolerogenic dendritic cell (DC) phenotype and expanding Tregs in vivo. This occurs through the activation of DCs’ GPR120 receptor signaling with an up-regulation of SOCS3 and a subsequent down-regulation of the JAK2-STAT3 signaling pathway [50]. However, despite promising preclinical and observational evidence, clinical trials investigating PUFA supplementation in humans have yielded inconclusive results [51].

Trans fatty acids (TFAs) are a subclass of unsaturated fatty acids that contain two or more unconjugated double bonds in the trans configuration. Although a small proportion of TFAs are naturally produced during ruminal fermentation in ruminant animals, most of them are generated industrially through the partial hydrogenation of PUFA-rich vegetable oils [52]. Dietary intake of TFAs has been associated with chronic metabolic disorders. In experimental models of obesity, TFA administration was found to induce gut microbiota dysbiosis, characterized by a slight increase in relative abundance of Proteobacteria and in the genus *Bacteroides*, accompanied by a decrease in Muribaculaceae, compared with control animals [53]. Similarly, a high-TFA, high-sucrose diet in mice led to an increased abundance of the *Desulfovibrionaceae* family, concomitant with worsening of diabetes and hepatic steatosis [54].

Although no preclinical or clinical studies have yet specifically investigated the effects of TFA intake on MS pathology, this body of evidence from metabolic disease models suggests a role of TFA in inducing gut dysbiosis also in MS. TFAs are typically present in ultra-processed foods (UPFs) in varying amounts depending on the degree of industrial processing [55]. Data from the 2003–2006 Ausimmune Study revealed that higher UPF consumption was significantly associated with an increased likelihood of a first demyelinating event, with an 8% increase for an energy/day adjusted portion of UPF [56]. Moreover, individuals with higher UPF intake exhibited moderate-to-severe MS disease activity compared with those consuming lower amounts, both in unadjusted analyses and after adjustment for demographic and clinical confounders [57].

High-fat (HF) diets have produced intriguing results in EAE, highlighting the importance of dietary fat quality. In one study, EAE-induced mice fed a HF diet based on canola oil, which is rich in MUFAs and contains approximately 30% PUFAs, showed complete protection from disease, exhibiting no clinical signs and zero incidence compared with mice fed high-carbohydrate or high-protein diets. Furthermore, HF-fed mice displayed limited CNS infiltration and no evidence of demyelination [58]. These findings suggest that specific types of dietary fats, rather than total fat content, may exert protective effects against MS-like pathology. Mechanistically, LCFAs, such as lauric acid, have been shown to promote pro-inflammatory cytokine production (including IFN-γ, IL-17A, and IL-2) in CD4^+^ T cells and drive Th17 differentiation, thereby exacerbating disease severity in EAE [35,59]. In contrast, OA not only stimulates T cell proliferation in lymphoid organs but also suppresses IL-2 and IFN-γ production [60]. Additionally, OA alters cell membrane composition by reducing arachidonic acid levels, resulting in decreased production of pro-inflammatory lipid mediators derived from arachidonic acid [61]. In pwMS, adipose tissue OA levels are lower than in healthy controls, and ex vivo treatment of Tregs from pwMS with OA partially restores their suppressive function [62]. Polyunsaturated fatty acids also demonstrate beneficial effects in MS and EAE, too. Linoleic acid, an omega-6 fatty acid comprising roughly 19% of canola oil, has been reported to modulate inflammatory responses in both preclinical and clinical studies [63,64]. Overall, fatty acids exert several opposing effects on MS pathology and are summarized in Table 1. While certain medium- and long-chain fatty acids promote pro-inflammatory responses, SCFAs, some MUFAs and omega-3 show immunoregulatory and neuroprotective properties. TFA and UPF, instead, appear to worsen inflammation and dysbiosis. Emerging evidence also points to specific HF dietary patterns, such as canola oil-based diets, as potentially protective. In conclusion, although their role in MS is variable, fatty acids remain essential dietary components that, when consumed in balance, can also contribute to general health benefits.

**Table 1:** List of the different biological and clinical effects reported for fatty acids intake on EAE and MS. Chain length:double bonds (C:N), Saturated fats (SF), C:N = (chain length:double bonds).

References	Dietary Fatty Acids	C:N	Pathology	Biological Effects	Clinical Effects
Haghikia et al., 2015 [35]	Lauric acid	SF, C12:0	EAE	**↑** Differentiation Th1 and Th17 cells; **↑** Th17 in spleen and CNS; Gut microbiome involvement;	Worsening disease course
Haghikia et al., 2015 [35]	Palmitic acid	SF, C16:0	EAE	**↑** Differentiation Th1 and Th17 cells	Worsening disease course
Conde et al., 2020 [39]; Conde et al., 2019 [40]	Oleic acid—EVOO	MUFA, CIS-18:1 (ω-9)	EAE	**↑** Lipid peroxidation; **↓** Carbonylated proteins; **↓** Glutathione peroxidase; **↓** Brain, spinal cord, blood and intestine levels of TNF-alpha, NF-kBp065 and NO; **↓** Serum and intestinal LPS	Improved clinical parameters
Ni et al., 2025 [58]	Oleic acid linoleic acid— Canola oil	MUFA, CIS-18:1 (ω-9) PUFA, 18:2 (ω-6)	EAE	Limited CNS infiltration and no evidence of demyelination compared to high carbohydrates and high proteins diet	Zero incidence and clinical score
Feng et al., 2021 [50]; Adkins et al., 2019 [47]; Unoda et al., 2013 [48]; Ouyang et al., 2020 [49]	EPA, DHA	PUFA, 20:5 (ω-3) PUFA, 22:5 (ω-3)	EAE	**↓** Phosphorylated transforming growth factor β-activated kinase 1, inhibition terminal activation of NF-κB; **↓** T-cell activation; Anti-inflammatory effect, induction of a tolerogenic DC phenotype with increased Tregs	Reduce clinical severity
Langer-Gould et al., 2020 [43]	EPA, DHA	PUFA, 20:5 (ω-3) PUFA, 22:5 (ω-3)	MS		Consuming fish/seafood at least once a week or at least once a month with regular fish oil use associated with 44% reduced odds of MS/CIS
Bjørnevik et al., 2017 [42]	ALA	PUFA, 18:3 (ω-3)	MS		Associated with a lower risk of MS

**↑** indicates increase; **↓** indicate decrease.

## 4. Dietary Carbohydrates

Carbohydrates, commonly referred to as sugars, are one of the essential macronutrients in human nutrition. They represent a major source of energy and play a key role in the regulation of glucose and insulin metabolism, as well as in the modulation of triglyceride and cholesterol levels. Moreover, carbohydrates are substrates for fermentation processes that occur within the gut microbiota [65].

Carbohydrates can be further classified into simple carbohydrates, complex carbohydrates, starches, and dietary fibers. The latter, due to their specific physicochemical properties and well-documented health benefits, will be discussed in detail in the following section. Given the biochemical diversity of this macronutrient group and the wide range of physiological pathways in which they participate, carbohydrates make a substantial contribution to overall human health.

In PwMS, a lower intake of total carbohydrates and sugars has been associated with improved walking ability and a lower degree of disability [66,67]. Conversely, individuals consuming diets rich in simple carbohydrates but insufficient in total carbohydrate and thiamine relative to their nutritional needs exhibit higher levels of MS-associated depression [68].

A high intake of carbohydrates has been linked to the promotion of a pro-inflammatory metabolic environment [69]. Accordingly, several studies have explored the relationship between their consumption and the risk of developing or exacerbating chronic inflammatory and autoimmune diseases, including MS.

Studies conducted in EAE mice have demonstrated that the consumption of caffeine-free high-sucrose (10–11% *w*/*v*) cola beverages for a long period of 8 weeks can exacerbate disease pathogenesis through a microbiota-dependent mechanism, involving the expansion of Th17 cells. Specifically, this dietary intervention favored the growth of several bacterial genera, including *Mucispirillum*, *Coprococcus*, *Escherichia*, *Paraprevotella*, *Desulfovibrio*, *Odoribacter*, and *Ruminococcus*, as well as the species *Escherichia coli*, *Ruminococcus gnavus*, *Mucispirillum schaedleri*, *Desulfovibrio C21_c20*, and *Butyricicoccus pullicaecorum* [70].

A similar alteration in gut microbial composition was observed in another EAE model fed with a fructose-rich diet (649.19 g/kg or 70% kcal from fructose, in some cases supplemented with 30% *w*/*v* fructose-containing water). This diet significantly reduced the abundance of beneficial commensals while favoring the proliferation of pro-inflammatory bacteria. Although the high fructose intake had only a modest effect on disease severity, an immune modulation was observed both in the gut and periphery. In particular, an enrichment of Helios^−^RORγt^+^Foxp3^+^CD4^+^ Treg cells was detected in the small intestinal lamina propria [71] probably as a result of fructose absorption in the small intestine [72]. The expansion of Helios^−^RORγt^+^Foxp3^+^CD4^+^ Tregs may appear to contrast with the pro-inflammatory effects typically attributed to high fructose consumption; however, the functional nature of this subset remains unclear. Helios is generally considered a marker of thymus-derived Tregs, while the observed Helios^−^ populations likely represent peripherally induced subsets shaped by environmental factors such as diet and microbiota composition [73,74,75,76]. Moreover, RORγt serves as the lineage-defining transcription factor for Th17 cells; thus, Tregs co-expressing Foxp3 and RORγt may represent IL-17-producing Tregs with reduced suppressive capacity [77,78]. Nonetheless, other reports indicate that these double-positive Tregs can retain significant immunosuppressive properties [79]. Taken together, these findings suggest that the presence of this specific Treg subset within the small intestinal lamina propria could be diet-dependent, potentially induced by high fructose intake through gut microbiota remodeling, although its precise immunological function in inflammatory contexts remains to be fully elucidated.

Further evidence on sugar-mediated inflammation in EAE was provided by Zhang et al., who demonstrated that the administration of a high-glucose diet (20% glucose for 2 weeks) induced the generation of reactive oxygen species (ROS), which modulate transforming growth factor beta (TGF-β) signaling, thereby promoting Th17 cell differentiation and their accumulation within the spinal cord and brain [80].

More recently, a 2025 study reported that a high-carbohydrate diet (75% carbohydrates for 6–7 weeks) was associated with an earlier onset of EAE, a higher disease incidence and with more severe clinical symptoms. Mice fed this diet displayed higher CNS immune cell infiltration and pronounced demyelination, indicating that excessive carbohydrate consumption acts as an aggravating factor in EAE pathogenesis [58].

Overall, these findings suggest a potential association between high carbohydrate intake and MS. However, it is important to consider the source and quality of carbohydrates. The studies discussed mainly address the detrimental effects of simple sugars present in sugar-sweetened beverages and ultra-processed foods. Conversely, a diet providing an appropriate proportion of carbohydrates (approximately 50–55% of total energy), primarily derived from whole grains and unprocessed or minimally processed foods such as fresh fruit, is recommended to ensure balanced macronutrient intake. Such dietary patterns are associated with a higher intake of fiber and micronutrients and a lower risk of mortality [81]. Therefore, the adoption of anti-inflammatory dietary patterns appears to offer promising opportunities as an adjunct to conventional therapies for MS management. Nevertheless, further evidence from well-designed studies is required to substantiate these findings and clarify the mechanisms underlying their potential benefits. The main effects of dietary carbohydrates on EAE and MS are summarized in Table 2.

**Table 2:** List of the different biological and clinical effects reported for carbohydrates intake on EAE and MS.

References	Dietary Carbohydrates/Diet	Phatology	Biological Effects	Clinical Effects
Zhang et al., 2019 [80]	Glucose (20% of glucose-diet)	EAE	**↑** Differentiation of Th17 cells, elevated into the spinal cords and brain; **↑** ROS; Activation TGF-β	Higher disease severity
Peterson et al., 2023 [71]	Fructose (70% of diet)	EAE	Modulation of gut microbiota composition; **↑** Helios−RORγt + Foxp3 + CD4^+^ Treg cells appeared in the small intestine lamina	Minimal influence on the EAE severity
Cao et al., 2017 [70]	Sucrose (10–11% *w*/*v* of cola beverages)	EAE	**↑** Th17 cell Microbiota-dependent mechanism	Exacerbate disease pathogenesis
Ni et al., 2025 [58]	Carbohydrates (75% of diet)	EAE	**↑** Infiltrating CD4^+^, CD8^+^ and RORγt + CD4^+^ T cells and inflammatory macrophages; Exacerbated neuroinflammation and peripheral T cell inflammatory cytokine responses	Earlier EAE onset; **↑** Disease incidence; **↑** Maximum and cumulative clinical scores
Bromley et al., 2019 [66]; Fitzgerald et al., 2018 [67]	Lower intake of carbohydrates and sugars	MS		Improved walking ability; lower disability
De la Rubia Ortí et al., 2020 [68]	Simple carbohydrate diet	MS		**↑** Levels of MS-associated depression

**↑** indicated increase.

## 5. Dietary Fibers

Dietary fibers are indigestible carbohydrates derived from plant sources. They are generally classified as soluble or insoluble, and as fermentable or non-fermentable complex carbohydrates. Fibers are naturally present in a wide range of commonly consumed foods, including fruits, grains, vegetables, nuts, legumes, and seaweeds, although their content varies depending on the source [82,83]. The impact of both fermentable and non-fermentable fibers has been explored in the context of MS, as higher dietary fiber intake has been associated with significant improvements in disease outcomes compared with a Western-type diet [84]. The main effects of dietary fiber on EAE and MS are summarized in Table 3.

The end products of fermentable fibers are the SCFAs, whose protective effects against MS have been extensively documented. Fettig et al. demonstrated that guar gum, a soluble and fermentable fiber, significantly reduced the activation and migration of CD4^+^ Th1 cells, resulting in attenuated neuroinflammation following EAE induction [85]. In contrast, non-fermentable fibers, commonly found in vegetarian diets, have been investigated for their immunomodulatory effects despite their limited degradation by gut microbiota [83]. A diet enriched in non-fermentable fibers (cellulose-rich, CR diet) was shown to prevent disease development in EAE mice. This protective effect was linked to an increased production of LCFAs, which promoted a Th2 anti-inflammatory immune response and modulated gut microbial composition [86].

Other findings supporting the protective role of dietary fibers in EAE come from studies on oral administration of pomegranate peel extract, as pomegranate peel is composed primarily of soluble and insoluble fibers in nearly equal proportions [87]. This treatment has been shown to ameliorate EAE, by reducing CNS infiltration and preventing myelin loss, and by modulating gut microbiota composition [88].

Conversely, Sen et al. reported that a high-fiber diet was associated with a pro-inflammatory milieu in EAE, leading to a worsened disease course compared with a fiber-free diet [89]. Peripheral blood cells displayed an enhanced pro-inflammatory phenotype, accompanied by increased infiltration of dendritic cells and monocytes into the CNS. Notably, this inflammatory environment was observed despite the increase in SCFA levels, highlighting the context-dependent and potentially bifunctional nature of these metabolites as deeply discussed in the following section [89].

In line with preclinical data, several human studies have investigated the role of dietary fiber intake in MS. Since 1998, numerous studies have examined the potential protective effect of a high-fiber diet on MS risk [90], as it has been observed that pwMS often consume less fiber than recommended [91]. A low fiber intake has been associated with an increased risk of a first demyelinating event [92], whereas a diet enriched in prebiotic fibers was shown to attenuate systemic inflammation and modulate disease severity in pwMS [93]. Similarly, higher fiber consumption and greater adherence to the MD have been linked to lower Multiple Sclerosis Severity Scores (MSSSs) and improved clinical outcomes [94,95]. Conversely, Hatami et al. reported a positive association between total fiber intake and the odds of developing MS [96].

### Gut Microbiome Modulation by Dietary Fibers and Their Products SCFA

The health benefits associated with a high-fiber diet may be attributed, at least in part, to its capacity to modulate gut microbiota composition and activity. For instance, intake of pomegranate peel extract, rich in both soluble and insoluble fibers, has been shown not only to ameliorate EAE outcomes but also to significantly influence gut microbiota composition, increasing the relative abundance of *Lactobacillaceae* and reducing *Alcaligenaceae* and *Acidaminococcaceae* families [88]. Similarly, an aqueous extract of *Palmaria palmata* demonstrated potential neuroprotective effects against cuprizone-induced demyelination, while promoting beneficial shifts in the gut microbiota, by promoting the growth of the beneficial Bacteroidia, *Lactobacillus* and Proteobacteria communities [97]. Moreover, a diet rich in non-fermentable fibers was reported to affect microbial alpha diversity, by significantly lowering in the cecal microbiota of CR-fed mice compared with controls. Also, beta diversity was found to be different between the two groups. At the taxonomic level, increases were observed in the families *Helicobacteriaceae*, *Ruminococcaceae*, and *Enterococcaceae*, while *Lactobacillaceae*, *Coriobacteriaceae*, and *Sutterellaceae* were reduced following CR diet administration. At the genus level, *Enterococcus*, *Helicobacter*, *Parabacteroides*, *Desulfovibrio*, *Pseudoflavonifractor*, and *Oscillibacter* were enriched, whereas *Parasutterella*, *Lactobacillus*, *Coprobacillus*, and *TM7 genera Incertae Sedis* were decreased [86].

**Table 3:** List of the different biological and clinical effects reported for fiber intake on EAE and MS.

References	Dietary Fibers/Diet	Pathology	Biological Effects	Clinical Effects
Fettig et al., 2022 [85]	Guar gum	EAE	**↓** Activation and migration of CD4^+^ Th1; delays in neuroinflammation onset	Delay EAE onset
Berer et al., 2018 [86]	Non fermentable fibers	EAE	**↑** LCFAs, which promoted autoimmune suppressive Th2 immune response; Gut microbiota modulation (**↑** *Enterococcus*, *Helicobacter*, *Parabacteroides*, *Desulfovibrio, Pseudoflavonifractor* and *Oscillibacter*, **↓** *Parasutterella*, *Lactobacillus*, *Coprobacillus* and *TM7 genera Incertae Sedis)*	Prevention of autoimmune disease
Lu et al., 2020 [88]	Oral pomegranate peel extract	EAE	**↓** CNS infiltration and myelin loss; Gut microbiota modulation (**↑** Prevotellaceae, **↓** Bacteroidales_S24_7)	Amelioration of EAE
Yousof et al., 2023 [97]	*Palmaria palmata* aqueous extract	Cuprizone-induced MS	Gut microbiota modulation (**↑** in Bacteroidia, *Lactobacillus* and Proteobacteria)	Protection against cuprizone-induced MS
Sen et al., 2023 [89]	High fiber intake	EAE	**↑** Proinflammatory environment; **↑** DC and monocytes infiltration in the CNS; **↑** SCFA	Worsening of general disease course
Mizuno et al., 2017 [98]	High fiber intake	EAE	Block of IFN-γ production; **↑** IL-17, Treg and macrophages	Amelioration of disease severity of autoimmune inflammatory diseases
Cavalla et al., 2022 [92]	Low fiber intake	MS		**↑** Risk of a demyelinating event
Moravejolahkamidoi et al., 2019 [93]	High prebiotic fiber intake	MS	**↓** Systemic inflammation	Modulation of disease severity
Bronzini et al., 2024 [94]	High fiber intake and adherence to MD	MS		**↓** MSSS
Marck et al., 2021 [95]	High fiber intake	MS		**↑** Health outcome
Hatami et al., 2024 [96]	High fiber intake	MS		**↑** Odds of MS

↑ indicates increase; ↓ indicates decrease.

In a pediatric cohort of pwMS, higher fiber intake was associated with an enrichment of *Clostridiales vadinBB60* and *Ruminococcaceae NK4A214* groups, as well as *Alistipes indistinctus YIT 12060*, while it correlated with a decrease in *Methanobrevibacter*, *Eggerthella* spp., *Lactococcus* spp., and *Anaerovoracaceae XIII AD3011* species. Interestingly, the reduction in these taxa was associated with an increased MS odds ratio for MS, whereas their enrichment appeared to confer a protective association, with the exception for *Anaerovoracaceae XIII AD3011* [99].

The effects of a high-fiber diet on the gut microbiota are mediated by the ability of specific bacterial taxa to ferment dietary fibers and produce SCFAs, metabolites extensively characterized for their immunomodulatory and neuroprotective properties [100]. In EAE models, SCFA supplementation suppressed astrocyte activation, suggesting a therapeutic potential for inflammatory CNS disorders [101]. Similarly, SCFAs derived from fiber fermentation, principally butyrate, propionate, acetate, and valerate, exert anti-inflammatory effects both locally within the gut and systemically, modulating Treg differentiation and BBB integrity in MS [102]. In EAE, oral supplementation with propionate or butyrate mitigates disease severity, increases Tregs, and reduces Th1/Th17 responses, largely through histone deacetylase (HDAC) inhibition and activation of GPR43 (FFAR2), GPR41 (FFAR3), and GPR109A (HCAR2) on intestinal antigen-presenting cells and T cells [103,104]. Locally, millimolar concentrations of SCFAs in the gut lumen promote epithelial barrier integrity and immune tolerance, whereas systemic micromolar levels engage G-protein-coupled receptors that can mediate either anti- or pro-inflammatory signaling depending on cell type and context [105,106]. In the CNS, butyrate and propionate have been shown to reduce microglial activation, preserve BBB integrity, and enhance remyelination in preclinical models [107,108], although the extent to which SCFAs directly reach or act within the human CNS remains uncertain.

Among SCFA, butyrate is predominantly produced by *Eubacterium*, *Clostridium*, *Bacteroides fragilis*, and *Faecalibacterium prausnitzii* through the fermentation of non-digestible fibers. Its effects are exerted on two fronts: within the gut, it contributes to gut homeostasis by promoting Treg differentiation and maintaining epithelial barrier integrity; in the CNS where it influences the permeability of the BBB and neuroinflammation [102]. Reduced butyrate levels, together with a depletion of SCFA-producing bacteria in gut microbiota, have been consistently observed in pwMS [109,110].

Propionate, mainly produced by *Bacteroidetes* and *Clostridia* species, also displays potent immunoregulatory functions through G-protein-coupled receptors such as GPR43, which enhances Treg differentiation [102]. Serum and stool concentrations of propionate are significantly reduced in pwMS compared to healthy controls [103].

Acetate, one of the most abundant SCFAs, is synthesized primarily by *Blautia hydrogenotrophica* and *Marvinbryantia formatexigens* within the *Firmicutes* phylum. Findings regarding acetate in MS are conflicting: elevated acetate levels have been associated with higher expanded disability status scale (EDSS) and MSSS values, along with an increase in IL-17-producing CD8^+^ T cells and a reduction in naïve CD4^+^ T cells [111]; however, other reports describe a beneficial association between acetate levels and disease activity [112]. Notably, acetate also promotes butyrate synthesis through a cross-feeding mechanism among gut commensals, suggesting a cooperative role in sustaining SCFA-mediated homeostasis [102].

Valerate, though less studied, is produced through the fermentation of branched-chain amino acids by *Prevotella stercorea*, *Prevotella copri*, *Mobilicoccus massiliensis*, and certain *Escherichia coli* strains. It has been shown to inhibit Th17 and IL-17A proliferation, ameliorate EAE severity, reduce CD4^+^ T-cell infiltration into the CNS, and enhance IL-10 secretion by B cells [102]. Nevertheless, pro-inflammatory effects of SCFAs have also been described in EAE, reflecting their dual and context-dependent role in modulating immune responses within the CNS [89,113].

The duality of SCFA was investigated by Mizuno et al. [98], who demonstrated that a high-fiber diet (30% pectin) ameliorated the severity of autoimmune diseases depending on the context of immune cell activation. In EAE and collagen—induced arthritis, SCFA treatment suppressed IFN-γ production while enhancing Treg cell levels, thereby exerting anti-inflammatory effects on adaptive immunity. By contrast, in antibody-induced arthritis, where innate immune responses predominate, SCFA exacerbated disease progression by promoting macrophage activation. These results suggest that while SCFAs exert anti-inflammatory effects in adaptive immunity-driven conditions such as EAE, they may conversely enhance innate immune responses, leading to inflammation in macrophage-mediated contexts. This dual mechanism could partly explain the increased monocyte infiltration into the CNS reported by Sen et al. [89].

Moreover, recent evidence indicates that they may also increase intestinal permeability, reduce mucus layer thickness, and elevate pro-inflammatory cytokines such as IL-17 [114]. Increased acetate levels have been detected in MS patients compared with healthy controls, correlating with higher EDSS and MSSS scores and with IL-17^+^ T-cell abundance [111]. Furthermore, butyrate and valerate concentrations were found to correlate positively with inflammatory mediators, including TNF, IFN-γ, and IL1R1 [112]. These findings suggest that in the context of EAE and MS, where gut dysbiosis and systemic inflammation are prevalent, higher levels of specific SCFA could potentially aggravate disease pathology, instead of promoting a protective environment. As discussed above, variability in dose, compartment exposure, and receptor engagement likely further underlie the mixed pro- and anti-inflammatory outcomes observed across studies. Future work should quantify local SCFA concentrations in intestinal and CNS compartments and dissect receptor-specific versus epigenetic mechanisms in human settings, could help in understanding the dual role of SCFA in MS.

Taken together, the evidence from experimental models and humans studies underscores the complex and context-dependent effects of dietary fibers and their metabolites on immune regulation and neuroinflammation. While fermentable fibers and their derivatives, such as SCFAs, can exert anti-inflammatory and neuroprotective effects, excessive fiber intake or specific SCFA profiles may instead promote inflammation, depending on the immune and microbial environment. However, overall findings underscore the predominantly protective role of dietary fiber in MS, further emphasizing the importance of a healthy diet in improving disease outcomes and paving the way for future dietary interventions as part of its management.

## 6. Dietary Proteins

In this context, beyond carbohydrates and fibers, dietary proteins also emerge as crucial modulators of immune and neuroinflammatory processes, influencing both gut microbial metabolism and CNS function. Proteins are essential macronutrients required for the maintenance and proper functioning of the human body. They are primarily obtained through dietary sources and play critical roles in numerous physiological and metabolic processes. In recent years, increasing attention has been devoted to understanding how dietary proteins may influence the course of MS. The following section will discuss current evidence regarding the impact of proteins derived from meat and dairy products on MS progression. Additionally, the potential roles of dietary tryptophan and gluten in modulating disease mechanisms will be explored. The main effects of dietary proteins on EAE and MS are summarized in Table 4.

### 6.1. Proteins from Meat

Meat, defined as the muscular tissue of animals, is typically classified by color into red and white meat. The red color derives from its high content of myoglobin, an iron-containing protein, found predominantly in pork, beef, lamb, and venison, compared to white meats such as chicken, turkey, and rabbit. Red meat represents a major source of proteins, saturated and unsaturated fats, vitamins, and minerals such as iron and zinc [115]. Processed red meat, including sliced meats, sausages, and hot dogs, is preserved by salting, flavoring fermentation, or the addition of chemical preservatives that enhance flavor, quality, and shelf life.

High consumption of animal-derived foods, particularly processed meats and excessive sodium intake, have been identified as major dietary risk factors for premature death and disability associated with non-communicable diseases including diabetes, cardiovascular diseases, cancer, and premature mortality [16,116,117]. However, when specific meat types are analyzed separately, different associations have been observed: while several reviews and meta-analyses have demonstrated a consistent positive association between red/processed meat consumption and mortality risk, the relationship between white meat intake and non-communicable disease risk remains less clearly established [118,119].

The adverse effects of red/processed meat may be attributed to components naturally present in raw meat, such as saturated fats and heme iron, or to compounds generated during cooking (e.g., advanced glycation end-products and nitrosamines) and processing (e.g., nitrites and salt). Among these, both iron and salt have been independently investigated in the context of MS.

With respect to iron, it is important to distinguish between heme and non-heme forms. Heme iron derives from animal products, while non-heme iron is found in both plant and animal foods. Although dietary iron intake contributes modestly to total body iron levels, most iron originates from erythrocyte recycling [120]. In MS, iron dysregulation has been reported, with accumulation in grey matter and depletion in normal-appearing white matter. This accumulation leads to cytotoxicity through oxidative stress, elevated proinflammatory cytokines, impaired DNA repair, and glutamate toxicity [121,122]. Despite serum iron concentrations being normal or slightly reduced in pwMS, ferritin levels are were found increased, suggesting altered iron storage and possible implications for dietary recommendations [120]. When Jumaylawee et al. investigated the association between heavy metal, as iron, present in the stool and gut microbiota in MS, they reported lower intestinal iron levels in pwMS compared to controls, accompanied by enrichment of *Lachnospiraceae*, *Ruminococcaceae*, and *Verrucomicrobiaceae* families, supporting the notion that metal homeostasis and gut microbiota interactions may be involved in MS pathogenesis [123].

Sodium chloride (NaCl) has also been linked to MS pathophysiology: elevated salt concentrations have been shown to enhance IL-17A production during Th17 cell differentiation in vitro and to promote the expansion of pathogenic Th17 subsets in vivo, contributing to increased MS incidence [124]. Salt effects are exerted also through gut microbiota, enriching proinflammatory genera such as *Megamonas*, *Parabacteroides*, and *Collinsella*, while depleting beneficial taxa such as *Lactobacillus* and *Faecalibacterium*, thereby increasing intestinal Th17 cell pathogenicity [125]. Confirming this, medium to high salt consumption in pwMS has been associated with higher relapse rates compared to low-salt intake [126].

Despite the evidence regarding these components, the direct association between red meat consumption and MS risk remains inconclusive, whereas data on white meat are currently lacking. Some studies found no association between red meat intake and MS risk [90,127], while others reported an increased risk in populations with higher red meat consumption in a more affluent socioeconomic status [128] or among individuals adhering to a meat-rich diet during youth [129]. Interestingly, Black et al. observed that unprocessed red meat consumption was associated with a reduced risk of a first demyelinating event, but only in women [130]. In contrast, processed meat consumption has been associated with an increased risk of MS in several studies [90,131,132], though not in others [127,130]. More recent findings indicate that adherence to a “prudent” dietary pattern, characterized by lower intake of red and processed meats, is linked to a reduced risk of MS compared to a Western dietary pattern [133,134,135].

Mechanistically, the potential link between red meat consumption and MS may be related to its potential effect on the immune system through the gut microbiome [136] or through molecular mimicry with dietary sialic acid derivatives, particularly the non-human glycan N-glycolylneuraminic acid (Neu5Gc) [137,138]. In a recent study in pwMS, higher meat consumption was associated with gut microbiota disturbances, with a reduced abundance of *Bacteroides thetaiotaomicron*, a fiber-fermenting bacterium negatively correlated with Th17 cell proportions and involved in methionine metabolism—an amino acid abundant in meat [136].

A second proposed mechanism involves Neu5Gc, a sialic acid found in most non-human mammals and enriched in red meats from cattle, pork, and lamb. Humans lack the enzyme cytidine monophosphate-N-acetylneuraminic acid hydroxylase (CMAH) required to synthesize Neu5Gc, resulting in exclusive expression of the homologous N-acetylneuraminic acid (Neu5Ac). Experimental studies have shown that dietary Neu5Gc can be incorporated into human tissues in trace amounts, effectively functioning as a dietary “xeno-autoantigen” [139,140]. Consequently, the production of anti-Neu5Gc antibodies has been proposed to contribute to chronic inflammation in humans, including MS [137]. Indeed, increased serum IgG reactivity toward both Neu5Gc and Neu5Ac has been detected in treatment-naïve MS patients [138]. Overall, elevated antibody responses to these sialic acid structures in pwMS may partly result from cross-reactive immune recognition, in which dietary exposure to Neu5Gc triggers autoimmune reactivity against self-antigens. Thus, increased red meat consumption, typical of Westernized diets, could represent a potential risk factor for MS in genetically or immunologically predisposed individuals.

### 6.2. Proteins from Dairy Products

The hypothesis of an association between milk and dairy product consumption and the risk of MS is not new, as it has been investigated for several decades, including studies exploring early-life exposure during adolescence [141,142]. This hypothesis is grounded in the concept of molecular mimicry, where an immune response initially directed against a foreign antigen cross-reacts with structurally similar self-antigens, potentially leading to autoimmune responses. According to this mechanism, the immune system could target specific milk-derived components that resemble CNS proteins.

The most abundant protein in bovine milk, the main type of milk consumed after weaning, is casein. Experimental studies demonstrated that mice immunized with casein developed demyelination associated with cross-reactivity toward myelin-associated glycoprotein (MAG), a key protein involved in myelin adhesion and stability [143]. Consistently, higher levels of anti-MAG antibodies have been detected in pwMS [144,145], and increased expression of casein family transcripts has been reported following inflammatory episodes in both EAE and MS [146]. These findings suggest that casein immunization could elicit autoimmune response against MAG through a molecular mimicry mechanism. Interestingly, pwMS with elevated antibody titers against β-casein were found to exhibit higher levels of disability [147].

Another milk component of interest is butyrophilin (BTN), one of the major constituents of the milk fat globule membrane, accounting for 20–45% of its total protein content in bovine milk [148]. BTN exhibits sequence homology with myelin oligodendrocyte glycoprotein (MOG), a transmembrane protein essential for myelin formation and maintenance of the myelin sheath. In Dark Agouti rats, immunization with BTN induced EAE through a T cell-dependent mechanism, with autoreactive T cells cross-reacting with both BTN and MOG, suggesting that initial exposure to BTN could trigger autoimmune responses against MOG [149]. Supporting this, experimental evidence showed that MOG-specific antibodies present in the cerebrospinal fluid of pwMS were able to cross-react with synthetic BTN peptides, an effect absent in healthy donors (HD) [150].

Other milk-derived components have also been implicated in MS onset, although they have been less extensively studied. One such molecule is monosialodihexosylganglioside (GM3), a ganglioside present in cell membranes and detectable in dairy products [151]. Sadatipour et al. reported that pwMS with primary and secondary progressive MS had higher levels of anti-GM3 antibodies compared to those with RRMS, HD, and individuals with other neurological diseases [152]. Another relevant compound is xanthine oxidase (XO), an enzyme abundant in milk that catalyzes the production of ROS and nitrogen species (RNS) [153,154]. Elevated levels of XO and associated ROS expression were detected in the CNS of EAE mice, reinforcing the established link between oxidative stress and the development of MS [155].

However, even if much of the literature identifies milk and its proteins as potential triggers of the autoimmune processes underlying MS, other studies have reported opposing effects. For example, Mañá et al. demonstrated that treatment of C57BL/6 mice with BTN, either before or after MOG immunization, prevented EAE development. BTN administration reduced Th1-associated proinflammatory cytokines, enhanced IL-10 secretion, and alleviated transient clinical symptoms [156]. Similarly, another study reported that administration of casein and lactose improved clinical outcomes and reduced inflammation and oxidative stress in EAE mice [157]. Furthermore, a population-based study conducted in Iran found an inverse association between dairy product consumption and MS risk [158]. At the same time, some studies have found no association between dairy intake and MS risk or demyelinating events [127,159].

Currently, knowledge regarding the effects of dairy consumption on the gut microbiota in MS remains limited. Ibrahim et al. investigated the effects of camel milk supplemented with *Bacillus amyloliquefaciens* in a MOG-immunized C57BL/6J mouse model. This intervention significantly reduced inflammatory cytokine expression and EAE severity while increasing total microbial load and SCFA levels [160].

In conclusion, several milk-derived components appear to be implicated in MS onset, either through the presence of pro-inflammatory molecules or via mechanisms of molecular mimicry. However, given the conflicting evidence on the relationship between dairy product consumption and MS risk, further studies are warranted to clarify the role of milk in disease pathogenesis and its potential impact on gut microbiota composition and immune regulation.

### 6.3. Proteins from Wheat

Wheat is one of the most widely cultivated crops worldwide, representing a major staple food and an important source of protein, vitamins, and dietary fiber. Gluten, the main protein fraction of wheat obtained after removing starch and water-soluble components from dough, has recently gained attention as one of the most incriminated dietary constituents. This is primarily because gluten is the key etiological factor in celiac disease, the most characteristic wheat-related disorder [161]. Despite current evidence not supporting a direct association between MS and celiac disease [162], gluten-free dietary interventions have been reported to improve EDSS scores, MRI lesion activity, fatigue perception, and quality of life in pwMS, although the number of available studies remains limited and the risk of bias is considerable [162].

Besides gluten, wheat also contains non-gluten carbohydrates and protein components, such as amylase/trypsin inhibitors (ATIs), which have been implicated in adverse immune-mediated reactions, including wheat allergy [163]. ATIs are a family of 17 structurally related gluten-free proteins that are abundant in wheat and act as natural defense molecules for the plant. These proteins are resistant to gastrointestinal enzymatic digestion and can activate myeloid cells in the intestinal lamina propria through Toll-like receptor 4 (TLR4) signaling [164,165].

Recent evidence suggests that ATIs may also promote inflammation within the CNS. In EAE, increasing dietary ATI content led to a worsening of clinical scores, accompanied by the detection of ATI-activated myeloid cell subsets in intestinal and extraintestinal sites, including the CNS. Specifically, ATIs enhanced the infiltration of pro-inflammatory CD45 + CD11b+ myeloid cells into the CNS and reduced the proportion of Foxp3 + CD25+ Treg cells in mesenteric lymph nodes. Interestingly, gluten alone did not elicit similar CNS inflammation [166]. In a small six-month bicentric proof-of-concept study, pwMS were randomized to follow either a standard wheat-containing diet for three months followed by a diet with less than 90% wheat content, or vice versa. While a reduction in circulating pro-inflammatory T cells was not observed with the lower ATI intake, a shift toward an anti-inflammatory monocyte phenotype was detected, accompanied by improved pain-related quality of life scores [167]. Although these findings require confirmation in larger, well-controlled clinical trials, they provide a basis for future dietary recommendations aimed at reducing ATI intake in pwMS, particularly during specific disease stages or as part of preventive strategies [161].

### 6.4. Tryptophan

Tryptophan is an essential amino acid obtained exclusively from the diet, found in foods such as meat, dairy products, legumes, and wheat. Its relevance in MS stems from its role as the biochemical precursor of serotonin, a neurotransmitter involved in affective and cognitive processes that are often impaired in pwMS. Reduced serum and platelet serotonin levels have been reported in pwMS, along with decreased availability of serotonin transporters in the limbic system of patients with RRMS [168,169,170].

The interest in the role of tryptophan in MS dates back to 1975, when Hyyppä et al. observed that pwMS treated with tryptophan for 30 days experienced improvements in motor function, bladder disturbances, and mood [171]. More recently, dietary tryptophan supplementation was shown to enhance memory performance in pwMS, although no significant effects on mood were detected [172].

These findings are now interpreted in the context of the gut–brain axis, whereby tryptophan metabolism by commensal bacteria gives rise to bioactive metabolites capable of modulating immune and neuronal function. In the EAE model, dietary tryptophan restriction impaired encephalitogenic T cell responses and significantly altered the gut microbiota composition [173]. Specifically, both α- and β-diversity were reduced in tryptophan-restricted mice, with marked decreases in the phyla *Bacteroidetes* and *Verrucomicrobia* and in the genera *Lactobacillus*, *Akkermansia*, and *Barnesiella*. Since these immunological effects were absent in germ-free mice, it was hypothesized that the modulation of T cell responses was mediated by the microbiota-dependent metabolism of tryptophan [173]. This provides a potential therapeutic avenue through microbiota-targeted dietary modulation of tryptophan metabolism in MS.

**Table 4:** List of the different biological and clinical effects reported for protein intake on EAE and MS.

Dietary Proteins	References	Pathology	Biological Effects	Clinical Effects
MEAT	Lauer et al., 1994 [128]; Gusev et al., 1996 [129]	MS		**↑** MS risk with higher red meat intake; **↑** MS with meat diet
	Black et al., 2019 [130]	MS		**↓** First diagnosis of CNS demyelination with unprocessed med in women
	Ghadirian et al., 1998 [90]; Lauer et al., 2007 [131]; Sepcić et al., 1993 [132]	MS		**↑** MS risk with processed meat
	Ghadirian et al., 1998 [90]; Zhang et al., 2000 [127]	MS		No association between red meat and MS
	Zhang et al., 2000 [127] Black et al., 2019 [130]	MS		No association between processed red meat and MS
	Black et al., 2019 [133]; Alfredsson et al., 2023 [134]; Veronese et al., 2022 [135]	MS		**↓** MS risk with prudent diet (low red/processed meat diet)
	Cantoni et al., 2022 [136]	MS	**↓** Microbiota fibre fermentation bacteria; **↑** Th17 cell	
DAIRY PRODUCTS	Otaegui et al., 2007 [146]	EAE/MS	**↑** Production of milk-related transcript of the casein family after inflammatory event	
	Escribano et al., 2022 [157]	EAE	**↓** Inflammation and oxidative stress with casein and lactose	Improve clinical aspect of the disease
	Chunder et al., 2023 [147]	MS	**↑** Ab titers against β-casein displayed greater levels of disability	
	Stefferl et al., 2000 [149]	EAE	Initial immunization with BTN triggers an autoimmune response against the MOG protein through a T cell-dependent mechanism	
	Mañá et al., 2004 [156]	EAE	**↓** Proliferation of pro-inflammatory Th1-related cytokines in response to MOG; **↑** IL-10 secretion in C57BL/6 mice	Prevention of EAE development; Alleviate transient clinical symptoms
	Sadatipour et al., 1998 [152]	MS	**↑** Levels of anti -GM3 in PPMS and SPMS	
	Honorat et al., 2013 [155]	EAE	**↑** XO levels and ROS expression in the CNS	
	Abbasi et al., 2017 [158]	MS		Reduction in the risk of MS
	Zhang et al., 2000 [127]; Dieu et al., 2022 [159]	MS		No association with MS risk or demyelination
	Ibrahim et al., 2023 [160]	EAE	**↓** Expression of inflammatory cytokines; **↑** Total microbial load; **↑** Levels of SCFAs in MOG-immunized C57BL/6J mouse model	Reduction in EAE disease index
WHEAT	Thomsen et al., 2019 [162]	MS		Gluten-free dietary interventions improved EDSS, MRI lesion activity, perceived fatigue and quality of life in MS patients
	Zevallos et al., 2023 [166]	EAE	Activation of myeloid cell; **↑** Pro-inflammatory CD45 + CD11b+ myeloid cells infiltrating the CNS; **↓** Foxp3 + CD25+ Treg cells in mesenteric lymph nodes	Worsening of EAE clinical scores with increasing amounts of dietary ATIs
	Engel et al., 2023 [167]	MS	**↑** In anti-inflammatory monocytes	Improvement in pain-related quality of life
TRYPTOPHAN	Sonner et al., 2019 [173]	EAE	Impaired encephalitogenic T cell responses due to tryptophan restriction; Modulation in gut microbiota; Impact on T cell responses mediated by gut microbiota	
	Hyyppä et al., 1975 [171]	MS		Amelioration in MS symptoms as motility, bladder disturbances and patients’ mood
	Lieben et al., 2018 [172]	MS		Amelioration in memory processes

↑ indicates increase; ↓ indicates decrease.

## 7. Clinical Implications of Dietary Intervention

Based on the evidence linking macronutrient composition to immune and metabolic homeostasis, several dietary patterns have been developed and evaluated for their potential impact on MS progression. Each of these regimens modulates nutrient intake differently, thereby influencing inflammatory and neurodegenerative processes.

**Figure 1:**
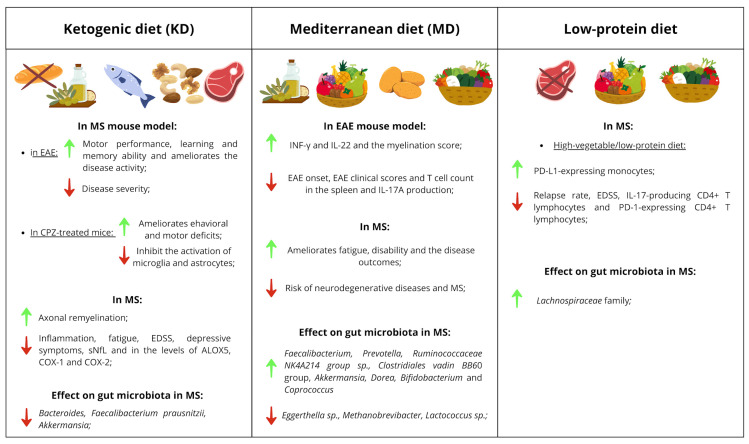
Tables of clinical implications of KD, MD and low-protein diets. The effects of these diets are divided into effects observed in mouse models (EAE and CPZ-treated mice), in MS patients and in the gut microbiota of pwMS. Green arrows indicate a positive effect of the diet, while red arrows indicate a negative effect.

One of the most investigated approaches is the ketogenic diet (KD), which aims at reducing pro-inflammatory consequences of carbohydrate intake (Figure 1). In this nutritional regimen, the reduction in carbohydrate intake is compensated by an increased consumption of fats and an adequate intake of proteins [174]. KD enhances mitochondrial function by increasing energy output and reducing ROS production, while elevating circulating ketone bodies. These metabolic adaptations contribute to reduced inflammation and neuroprotection [175]. In EAE, KD has been shown to decrease disease severity and improve motor, learning, and memory performance [176,177]. In the cuprizone-induced demyelination model, KD ameliorated behavioral and motor deficits and inhibited microglial and astrocytic activation, thus exerting neuroprotective effects [178]. Studies in pwMS have also documented beneficial outcomes associated with the adoption of this dietary regimen: in pwMS, KD has been associated with reduced inflammation, fatigue, disability, and depressive symptoms, along with enhanced remyelination and lower serum neurofilament light chain (sNfL) levels, a marker of neuroaxonal damage [177,179,180,181,182,183]. KD has also been reported to reduce the levels of enzymes involved in the synthesis of pro-inflammatory eicosanoids in pwMS. These eicosanoids play a key role in the pathogenesis of MS, as they contribute to increase vascular permeability and promote leukocyte migration into the CNS. Specifically, a reduction in ALOX5 expression was observed in pwMS compared to the HD group, and a down-regulation of COX-1 and COX-2 after KD, indicating an impaired activity of these enzymes following dietary intervention [184]. With respect to gut microbiota modulation, whereas KD initially reduced the abundance of several commensal bacteria such as *Bacteroides* and *Faecalibacterium prausnitzii* in pwMS, after 12 weeks, their levels progressively recovered to values comparable to healthy controls. Conversely, *Akkermansia* showed a slight but consistent decline throughout the intervention [185].

The MD represents another dietary pattern of interest especially for high fibre intake. MD is characterized by a high intake of plant-based foods such as vegetables, fruits, cereals, legumes, nuts, and olive oil, together with moderate consumption of dairy products, eggs, fish, and poultry, and limited red meat intake [186] (Figure 1). Adherence to MD has been consistently associated with lower risk of neurodegenerative diseases, including MS [186,187,188]. In pwMS, higher MD adherence correlated with reduced fatigue, disability, relapse risk, and the general disease outcomes [94,189,190,191,192,193,194,195,196]. MD also promotes favorable changes in the gut microbiota of pwMS, increasing the abundance of beneficial genera such as *Faecalibacterium* and *Prevotella* [197]. Furthermore, adherence to MD was found to be inversely associated with *Eggerthella* sp., *Methanobrevibacter* and *Lactococcus* sp., and positively associated with *Ruminococcaceae NK4A214 group* sp. and *Clostridiales vadin BB60 group* in MS [99]. In EAE, MD combined with lycopene delayed disease onset and reduced clinical scores, splenic T cell counts, and IL-17A production, while increasing IFN-γ, IL-22, and myelination scores [198]. Probably through action on gut microbiota: increased abundances of *Akkermansia*, *Dorea*, *Bifidobacterium*, and *Coprococcus* were found in mice fed with MD supplemented with lycopene compared to mice following a western diet [198].

Although high protein intake, especially from red meat, has been linked to pro-inflammatory effects, data on the impact of low-protein diets in MS remain limited (Figure 1). In 2017 a study evaluated the impact of a high-vegetable/low-protein diet in a cohort of patients with RRMS over a 12-month period, observing both clinical and cellular as well as microbiota-level changes [84]. Specifically, during the 12-month follow-up, a significant reduction in both relapse rate and EDSS was reported. At the immunological level, a significant decrease in IL-17-producing CD4^+^ T lymphocytes and PD-1-expressing CD4+ T lymphocytes was observed, alongside a significant increase in PD-L1-expressing monocytes. The proposed mechanism underlying these effects involves modulation of the gut microbiota, as the dietary intervention also led to a significant increase in the abundance of the *Lachnospiraceae* family [84]. 

Finally, growing attention has been directed toward vegan diets, characterized by the exclusion of all animal-derived products and reliance on plant-based protein sources. In a pilot study including patients with various neurological and psychiatric conditions, adherence to an anti-inflammatory vegan diet for three months led to improvements in cognition, energy, and activity levels in the MS participant [199]. Similar benefits were also reported in individuals with other neurodegenerative diseases such as Alzheimer’s and Parkinson’s disease, including better sleep quality, reduced pain, and enhanced physical activity [199]. While preliminary, these findings suggest that plant-based, anti-inflammatory vegan diets may exert beneficial effects on MS-related symptoms and warrant further investigation.

## 8. Discussion

Diet profoundly affects gut microbiota composition and the production of microbial metabolites, which in turn modulate immune responses relevant to MS pathogenesis. In this section, we summarize and critically discuss the most significant findings reported in our review.

LCFAs are a chemically heterogeneous group, encompassing SFAs, MUFAs, and PUFAs, which can exert diverse effects on neuroinflammation. While TFAs are predominantly found in industrially processed foods and are associated with gut dysbiosis and systemic inflammation [52,53,54,55,56,57], MUFAs including oleic acid from EVOO have shown beneficial effects on gut barrier integrity and an indirect anti-inflammatory mechanism via microbiota modulation, due to its polyphenolic components [38,39,40]. Additionally, ω-3 PUFAs including EPA and DHA, have consistently shown anti-inflammatory effects across experimental studies [42,47,48,49,50]. This evidence supports the non-negligible role of LCFA in immune and neuroinflammatory pathways, potentially through microbiota-dependent mechanisms. Despite this biological rationale, clinical trials investigating the effects of specific fatty acid profiles in MS remain limited to clarify their therapeutic relevance in MS.

A high intake of carbohydrates has been associated with the establishment of a pro-inflammatory metabolic milieu. Experimental studies in EAE models indicate that high-sucrose and high-fructose diets exacerbate disease pathogenesis through microbiota-dependent mechanisms and immunomodulatory effects [70,71]. Likewise, high-glucose diets have been shown to promote ROS production, thereby favoring Th17 cell differentiation [80]. In pwMS, both the total carbohydrate intake and, more importantly, the quality and type of carbohydrates consumed play a key role in modulating disease-related inflammation. Increased consumption of simple carbohydrates has been linked to greater depressive symptoms associated with MS [66,67,68]. Therefore, the source and quality of carbohydrates should be carefully considered: high consumption of simple sugars, particularly those found in sugar-sweetened beverages and ultra-processed foods, contributes to a pro-inflammatory state; conversely, a dietary pattern characterized by an adequate proportion of complex carbohydrates from whole grains, fruits, and other minimally processed foods is recommended and may confer beneficial effects on overall health.

Dietary fibers are a fundamental component of human nutrition, and their intake has been consistently associated with beneficial effects in both EAE and MS [82,83,84]. The positive health outcomes linked to a high-fiber diet are largely attributed to its ability to modulate the gut microbiota, favoring the expansion of anti-inflammatory and beneficial bacterial taxa, which in turn leads to the increased production of SCFAs [100]. SCFAs, such as acetate, propionate, and butyrate, have been shown to exert immunomodulatory and neuroprotective effects, influencing peripheral and central immune responses as well as maintaining intestinal barrier integrity [102]. Nevertheless, the relationship between dietary fibers, microbiota-derived metabolites, and host immunity is complex. Emerging evidence suggests that the effects of fiber supplementation may depend on the type, fermentability, and dosage of the fibers, as well as on the baseline microbial composition of the host [103,104,105,106,107,108]. Moreover, excessive fiber intake or specific SCFA profiles may promote pro-inflammatory responses, underscoring the importance of a balanced microbiota–host interaction [89,113]. Future studies are necessary to clarify the mechanisms through which different fiber sources and microbiota configurations modulate immune function and neuroinflammation, with the goal of optimizing dietary interventions for MS prevention and management.

The relationship between meat consumption and MS remains controversial. Evidence regarding red meat intake is inconsistent, even if some studies reported a higher risk of MS among patients with greater consumption of red and processed meats [90,129,131,132]. The potential adverse effects of red or processed meat could come from its components, such as saturated fatty acids, heme iron and sodium, as well as compounds formed during high-temperature cooking or industrial processing [120,121,122,123,124,125,126]. Mechanistically, the link between red meat consumption and MS pathophysiology may involve modulation of immune responses via alterations in gut microbiota composition and function [136]. Another mechanism could involve molecular mimicry, whereby dietary sialic acid derivatives may elicit cross-reactive immune responses against host neural antigens [137,138]. Overall, current evidence does not allow for clear conclusions, given the heterogeneity of study designs, dietary assessment methods, and population characteristics. Future research should aim to clarify the specific effects of different meat types, preparation methods, and associated dietary patterns.

Several studies have reported that immune responses directed against milk-derived proteins may aggravate disease activity, while others have described anti-inflammatory or neuroprotective properties associated with specific dairy components [149,156]. These inconsistencies likely reflect substantial heterogeneity in experimental design, the type and source of dairy products investigated and the technological processes involved in their production, and interindividual differences in genetic background and environmental exposures that shape immune tolerance. One explanation for the potential detrimental effects of dairy involves molecular mimicry mechanisms, whereby structural similarities between milk proteins and CNS antigens could trigger autoimmune cross-reactivity [147]. At the same time, findings indicate that the gut microbiota may act as an intermediary between dairy intake and host immune modulation. In this context, the metabolic activity of microbial communities and the bioactive compounds generated during fermentation could critically determine whether dairy consumption exerts pro- or anti-inflammatory effects. Taken together, the current body of research highlights the need for a deeper understanding of how dairy products influence MS pathophysiology.

The role of wheat proteins in MS has also been debated. In particular, gluten-free diet has been associated with improvements in EDSS, MRI lesion activity, fatigue and quality of life MS [162]. However, wheat contains not only gluten but also proteins as ATIs, which have been recently associated with inflammatory processes within CNS [163]. The current knowledge remains limited, and the available studies often include small sample sizes, heterogeneous populations, and potential confounding factors such as concurrent dietary modifications or lifestyle interventions. Therefore, while the hypothesis that gluten or other wheat-derived proteins may influence MS disease activity is plausible, further well-controlled, large-scale clinical and mechanistic studies are still required.

Studies have reported that pwMS treated with tryptophan supplementation experienced improvements in motor function, bladder disturbances, mood and memory performance [171,172]. Additionally, in EAE models, dietary tryptophan restriction impaired immune responses and altered gut microbiota composition [173]. These findings can be interpreted within the complex interactions of the gut–brain axis, highlighting the role of tryptophan metabolism by commensal bacteria in generating bioactive metabolites that can influence both immune regulation and neuronal activity. This emerging evidence suggests a potential therapeutic opportunity through microbiota-targeted dietary modulation of tryptophan metabolism in MS, but further studies are still required.

Overall, distinct dietary components, from fats and carbohydrates to fibers and amino acids, can exert divergent effects on MS pathogenesis via the microbiota–metabolite–immune axis. However, inconsistencies across models highlight the need for dose-controlled, longitudinal human studies to delineate causality and identify microbiota-derived biomarkers predictive of dietary response.

## 9. Conclusions

There is growing scientific interest in the role of diet due to its potential impact on health and the course of MS. Several studies have investigated the effects of diet and individual macronutrients on disease risk and progression, both in EAE and in MS. However, the majority of scientific evidence comes from animal models, while data in MS remains limited. Further targeted studies involving larger cohorts are needed to better understand whether certain foods may be more beneficial than others in promoting an anti-inflammatory environment with positive implications for disease outcomes. While it is unlikely that a single universally beneficial diet for MS patients can be identified, or that one macronutrient category can be recommended over others, clarifying the role of dietary components in relation to the disease could help to guide patients toward a healthy and balanced diet, with positive effects on their overall health. This would also support clinical practice, as physicians could recommend dietary patterns tailored to individual patient needs. Such an approach would not replace conventional therapies, but rather complement them, potentially enhancing both their efficacy and the patient’s quality of life.
